# Mapping the Relative Probability of Common Toad Occurrence in Terrestrial Lowland Farm Habitat in the United Kingdom

**DOI:** 10.1371/journal.pone.0148269

**Published:** 2016-02-03

**Authors:** Rosie D. Salazar, Robert A. Montgomery, Sarah E. Thresher, David W. Macdonald

**Affiliations:** 1 Wildlife Conservation Research Unit (WildCRU), Department of Zoology, University of Oxford, The Recanati-Kaplan Centre, Tubney House, Abingdon Road, Tubney, Oxfordshire, OX13 5QL, United Kingdom; 2 Department of Fisheries and Wildlife, Michigan State University, 480 Wilson Road, Room 13 Natural Resources Building, East Lansing, MI 48824, United States of America; Trier University, GERMANY

## Abstract

**Introduction:**

The common toad (*Bufo bufo*) is of increasing conservation concern in the United Kingdom (UK) due to dramatic population declines occurring in the past century. Many of these population declines coincided with reductions in both terrestrial and aquatic habitat availability and quality and have been primarily attributed to the effect of agricultural land conversion (of natural and semi-natural habitats to arable and pasture fields) and pond drainage. However, there is little evidence available to link habitat availability with common toad population declines, especially when examined at a broad landscape scale. Assessing such patterns of population declines at the landscape scale, for instance, require an understanding of how this species uses terrestrial habitat.

**Methods:**

We intensively studied the terrestrial resource selection of a large population of common toads in Oxfordshire, England, UK. Adult common toads were fitted with passive integrated transponder (PIT) tags to allow detection in the terrestrial environment using a portable PIT antenna once toads left the pond and before going into hibernation (April/May-October 2012 and 2013). We developed a population-level resource selection function (RSF) to assess the relative probability of toad occurrence in the terrestrial environment by collecting location data for 90 recaptured toads.

**Results:**

The predicted relative probability of toad occurrence for this population was greatest in wooded habitat near to water bodies; relative probability of occurrence declined dramatically > 50 m from these habitats. Toads also tended to select habitat near to their breeding pond and toad occurrence was negatively related to urban environments.

## Introduction

The global amphibian extinction crisis has been well documented and species declines are primarily attributed to habitat loss and degradation [1,2,3]. According to the IUCN Red List, 32% of amphibians worldwide are threatened, with 21% of those recognised as Endangered or Critically Endangered [4]. Despite what its name may suggest, the common toad (*Bufo bufo L*.) is not nearly as common as it used to be. In light of recent dramatic and largely unexplained population crashes, the UK Biodiversity Action Plan (UK BAP) recognized common toads as a priority species in 2007 [5]. Since designation, the decline of this species has not abated (e.g. [6]) requiring renewed conservation action to prevent further declines. For effective conservation action it is critical that we identify the causes of common toad population declines, in particular the effect of the dramatic landscape changes and pond losses associated with agricultural conversion and intensification in the UK over the past century [7,8].

Of specific need is a detailed understanding of how common toads select resources in the landscape. Common toads have excellent potential for use as a model species for determining the effect of land use change on habitat suitability and connectivity as they are moderately mobile (e.g. reported maximum migration of up to 3621m [9]), and crucially are reliant on both terrestrial and aquatic environments. In the terrestrial environment summer migration distances of up to 1km are not unusual [10]. The recognised philopatry of toads [11,12,13] limits their expected occupancy of the terrestrial environment to within migration distance of their natal breeding pond. In the terrestrial phase common toads are particularly difficult to study given that they are largely nocturnal and tend to spend resting periods in hiding [14]. However, as toads spend large portions of their lives out of water andutilize the terrestrial environment when travelling between breeding ponds, foraging or hibernation [15], conservation management options must consider the effect of terrestrial habitat availability. Indeed, it has been demonstrated that toads’ presence in breeding ponds is more dependent on terrestrial habitat quality than pond quality [16,17].

Common toad terrestrial habitat selection has primarily been assessed via radio-tracking [18,19,20] and manual searching. Both techniques pose problems for resource selection studies because radio-tracking is restricted to the number of toads it is possible to track at any one time, the tags themselves are finite (i.e., limited battery life), and the tags themselves are potentially detrimental to each individual’s movement behaviour and feeding [21]. Though radio-tracking produces a wealth of data per individual (many fixes) these points tend not to be independent potentially injecting dependencies into the models built to explain the ecological patterns. Radio-tracking does however, allow for capture of small scale local movement, which for the common toad is driven by resource availability (e.g. prey density) and abiotic condition (e.g. temperature) [22]; factors that cannot readily be assessed using methods where only one fix per individual is obtained. Manual searching is also problematic as it is unlikely to return many finds and could easily be biased toward habitat types in which animals are more visible, or habitats that are associated with observer experience. For natterjack toads (*Bufo calamita*), Denton and Beebee [23] found that searching for amphibians in the terrestrial environment can have highly variable levels of success, and may be disruptive to other fauna and destructive for the habitats being searched.

Passive Integrated Transponder (PIT) tags are used in this study to both individually identify toads and to aid detection of individuals in the terrestrial environment. These tags are relatively inexpensive when compared with radio-tags, and the number of individuals that can be marked is limited only by population size and the ability of surveyors to catch animals. As use of PIT tags allows the study of many more individuals, problems with spatial autocorrelation common in radio-tracking studies [24] are largely avoided as recaptures can be identified as different individuals. However, as toads are recaptured during the day when toads are resting, the habitats used during this period may be subtly different to those used at night when toads are migrating and foraging [10,14]. The deleterious effects of PIT tags on both common toad and common frog (*Rana temporaria*) were found to be negligible when compared with dye marking [25] and no effect of PIT tags was shown on breeding or survival of frog species (e.g. golden bell frog, *Litoria aurea*) [26] or recapture rates or body condition in the spadefoot toad (*Pelobates fuscus*) [27]. Similarly, no effect on growth or survival of Plethodontid salamanders was found when PIT tags were used to aid detection of animals below ground (with a detection efficiency of 44%, far above detection rates when employing hand-capture methods [28]).

We deployed PIT tags on common toads in a terrestrial landscape to develop a population-level resource selection function (RSF) during the breeding season. To our knowledge this is the first study to use PIT detector technology on amphibians in a large terrestrial system (see [23] for a study of Pyrenean brook salamanders in streams and [24] for a study of subterranean detection of ambystomatid salamanders within 36m of a breeding pond). The objective of this analysis was to determine the environmental features selected by common toads and the importance of proximity to these features by developing a map detailing the predicted relative probability of occurrence throughout the landscape.

The environmental features we considered included distance to breeding pond as all individuals were initially captured from this point and so were expected to remain close to this origin and due to philopatry [11,12,13]return to the same pond each breeding season. Distance to water features (including ponds, rivers, and drainage ditches) was expected to be important as toads would need to maintain access to water during the terrestrial phase to avoid desiccation. Wooded habitat offers dense vegetation with opportunities for toads to protect against desiccation and hide from predators. Positive association between amphibian occupancy, abundance, and diversity within woodland habitat coverage and water bodies has been demonstrated in several studies [29,30]. We considered urban areas as the bare ground of manmade surfaces (e.g. tarmac, concrete) would be risky to use as they offer no cover and so would have an increased level of exposure to drying or freezing weather, predation and risk of injury/death by traffic [31]. Though roads are used for migration[10], this is likely due to ease of movement in these habitats [13]. The toad is commonly the victim of traffic collision [32], as such roads are understood to pose important barriers to their migration. In evaluating patch occupancy, the surrounding habitat availability has more explanatory power if portions of habitat separated from the patch by barriers are removed (i.e. barrier based buffers) rather than considering all habitat within a circular buffer [33]. Edge habitats (the interface between two different land use types) were expected to be used as they were often characterised by a two different vegetation heights which would provide an area easy to move through and that benefits from increased insolation, but is also close to cover. Finally, we considered the effect of proximity to arable farmland as toads would be exposed to chemicals such as pesticides and fertilisers in these areas, reduced water quality [34], lower food availability than in adjacent field margins [35] and greater predation risk due to reduced cover. Occurrence of toads in breeding ponds has been shown to be negatively associated with arable farmland [36].

## Materials and Methods

### Study area and PIT tagging

**Fig 1:**
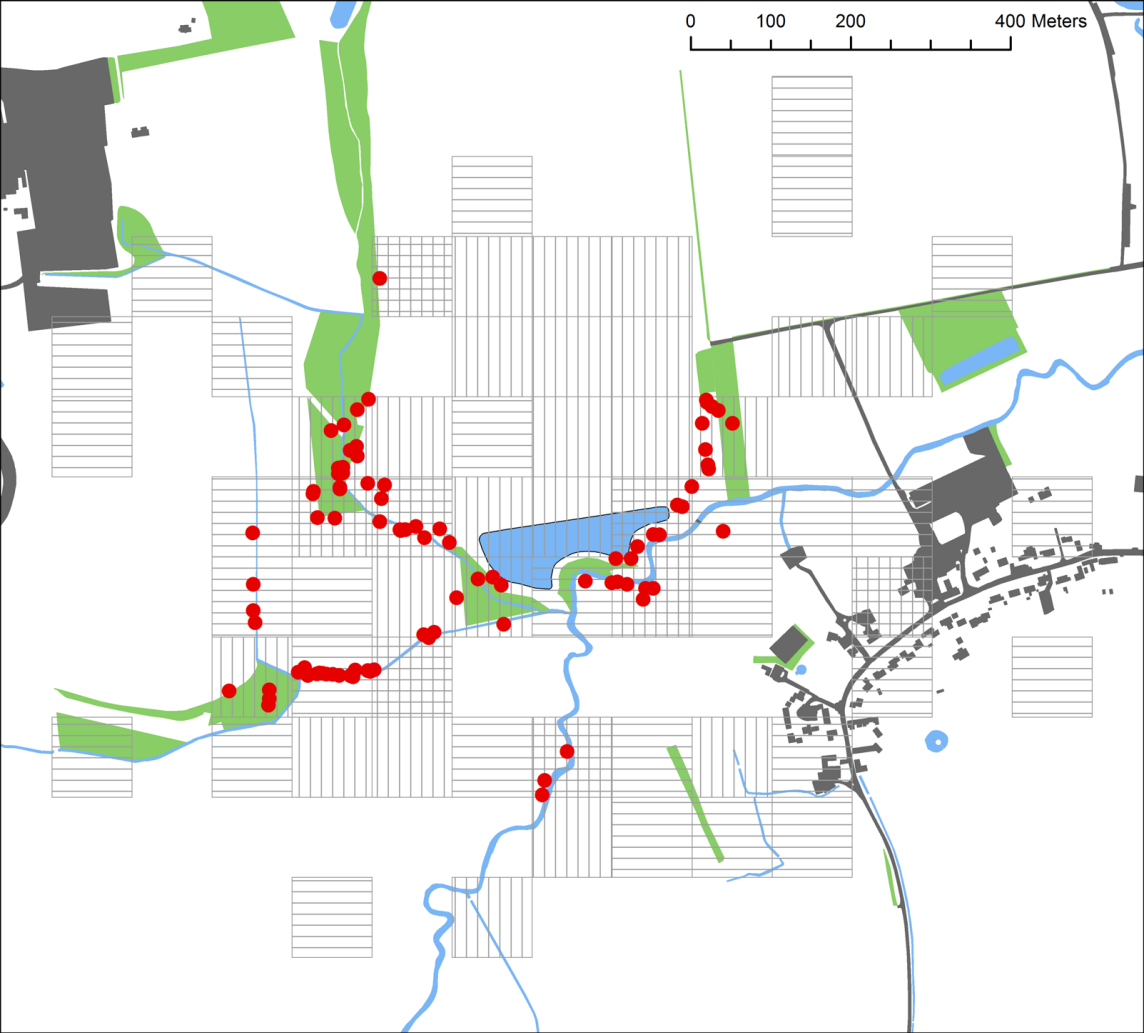
Characteristics of the landscape (green = wooded habitat, grey = urban areas, blue = water bodies) and PIT tag locations of common toads (red circles) in Garford, Oxfordshire (2012–2013). The breeding pond is outlined in black. Surveyed squares are shown in light grey (vertical hatch = 2012, horizontal hatch = 2013, cross hatch = surveyed both years).

We selected a constructed farmland pond known to have a large breeding population of common toads in Garford, Oxfordshire (Latitude, Longitude: 51.6633,-1.3919; Fig 1). This site, like many others in the larger agricultural matrix, represented a conversion of native forests and other natural habitats to farmland. The area surrounding this pond consists mainly of arable and pasture farmland, with patches of woodland, rough grassland, scrub, natural and constructed ponds, a small river and homes and gardens in the village of Garford.

During the breeding season (approximately 3 weeks from the beginning of March 2012 and 2 weeks from the beginning of April 2013) we captured toads at the Garford irrigation pond using visual search and hand nets. Passive Integrated Transponder tags (Trovan ID-100 FDX bio-compatible glass encapsulated tags, 128 kHz frequency, 11.5 x 2.12mm, 90mg) were injected subcutaneously (using a syringe implanter, each tag pre-sterilised and loaded in a disposable needle) into the pinched dorsum of 1040 adult toads in which snout to urostyle length (SUL) exceeded 50mm. As the injection site closed quickly and without intervention (we noted closed wounds in toads recaptured the day after tagging) we did not use either a suture or veterinary tissue adhesive following injection. Tag retention in common toads was reported by Brown [25] at 100% and similarly we had no incidence of capturing toads previously marked that had lost their tags. The bio-compatible glass casing of these tags prevents tissue irritation [37]. We recorded sex, weight, SUL (to the nearest mm) and PIT tag number, as well as the tag number of the other toad when toads were captured in amplexus (breeding position—female clasped around back by male). Following tagging, we released toads within 10m of capture locations. Toad capture and tagging continued until no toads were found in the pond after searches on three consecutive days.

### Ethics statement

Permission to conduct this study on private land was given by the landowners (Millets Farm, Garford and Garford village homeowners). All sampling procedures were reviewed and approved by the Central University Research Ethics Committee (CUREC). PIT tagging did not require a home office licence. We exceeded the minimum SUL (40mm) recommended by Pyke [26] to only tag toads with SUL>50mm. Experienced surveyors handled toads for the minimum length of time possible and kept them cool and shaded while awaiting tagging or measurement.

### Detection of toads in the terrestrial environment

Once tagging was complete (late March 2012, late April 2013), detection of toads began in the terrestrial environment using the portable PIT antenna and continued until first frost (October 2012 and 2013) when toads were expected to commence with hibernation. The PIT antenna used was a customised LID650 antenna and decoder box mounted on a carbon fibre pole. We passed the antenna over the ground, keeping it as close as possible to the base of the vegetation to allow reading through the vegetation and into the soil. We worked in teams of two, with one individual operating the detector and the other ensuring full coverage of the area, changing operators every 15 minutes to avoid fatigue. The antenna has a read range of between 17 and 36cm (dependent on tag orientation) and reads through water, soil, vegetation and air at the same rate [38]. We used ArcMap v10 to divide the study area into 100m by 100m numbered squares (Fig 1) to allow for random selection of squares for survey.

As it has been shown that it is important to consider detection probability when investigating the probability of occupancy of habitats [39], we next evaluated whether detection of the PIT tags was spatial biased by habitat type (particularly vegetation height and density). We deployed 234 PIT tags over the 23 trials (minimum 9, maximum 15 tags per trial) within the study area and our detection teams then attempted to relocate the tags. We repeated these trials 6–9 times per habitat type (short grassland (<20cm height); tall grass/herb (<1.5m height); woodland/scrub (ground vegetation <1.5m, woody vegetation/trees 2–20m). We found that detection rates across the three vegetation height classes were not significantly different (ANOVA, p>0.5, df = 2), thus we did not detect sampling bias associated with habitat type.

We searched a total of 40 squares in 2012 and 39 squares in 2013; each square took up to 6hrs to search thoroughly, depending on the area accessible. We searched within 500m of the breeding pond, as it was the maximum distance practically possible while maintaining a good probability of recapturing sufficient toads for modelling. The other consideration for this cut off distance was the ecology the common toad. For instance, the maximum migration distance (from the breeding pond to foraging area) reported for the common toad is approximately 3.6 km (see review, [9]) though studies have recorded migration much closer to the breeding pond, for example between 55m and 1600m in pine forest and pasture landscape of Bavaria, Germany [40], a maximum of 470m from the pond in upland Spain [19] and with maximum distances of between 170m and 1835m [41] away from the breeding pond in a mixture of agricultural and forest habitats in the Czech republic. We therefore secured access to the farmland and many gardens in the habitat at a 500m radius from the breeding pond. We operated the detector in all weather conditions possible as during the day toads are largely inactive [14] and so weather should not affect their behaviour during that period. We avoided stormy or highly windy conditions which considerably diminished our ability to hear the beep when a tag was detected. Once detected, toads were again sexed, weighed and measured. We then recorded the exact location using a hand held GPS unit.

### Environmental features

We developed a Geographic Information System (GIS) to describe the study area. This GIS consisted of a suite of covariates involving both aquatic and terrestrial features relevant to common toads during the breeding season. We calculated all covariates as distance metrics (in meters) from every portion of the study area to the nearest feature. Distance raster files were produced for distance to nearest wood, water body, urban habitat (manmade surfaces such as tarmac and concrete, buildings), arable farmland and edge habitat (i.e. boundary between two distinct land use types).

### Population-level resource selection function

We developed a RSF to estimate the proportional probability of common toad occurrence throughout the study area given a used-available design [42,43,44,45]. In this case, the locations returned from our PIT tag relocation functioned as our ‘used’ locations. We then needed to estimate ‘available’ locations in the landscape. To do so we randomly generated 4 locations per 100 m^2^ within the squares surveyed in each year of the study. We chose 4 locations per 100m^2^ because this procedure generated a sample of available locations at a ~3:1 ratio to used locations, which is a conventional ratio in RSF modelling (e.g. [46,47]). We ensured that no available location was generated within 20 m of a used location so that we did not confound the estimation of the probability of occurrence (e.g. [48]). Twenty meters is the extent of daily movements for common toads in the post-breeding season once they reach their foraging habitat [20,40]. These used and available locations became our binary response variable in a mixed-effects logistic regression model which we fit as a function of the environmental features in STATA 10 (StataCorp LP, College Station, TX). Given that we studied these toads over 2 consecutive breeding seasons (2012–2013) we modelled year of the study as a random intercept.

Our model selection procedures were based on established techniques for multivariate logistic regression models [49]. We initiated these procedures by calculating univariate models for each of the environmental features. We ranked variable importance using Wald statistics and retained those covariates that were deemed influential (i.e., *P* < 0.25; [49]). We next tested for colinearity using Pearson correlation coefficients. In cases were two covariates were correlated (|r| > 0.50), we eliminated the weakest predictor as determined by Wald statistics. This process resulted in a candidate covariate set without colinearity. Starting with the strongest predictor were developed a multivariate model in a stepwise fashion. The additional parameter was retained when a significant (α < 0.05) likelihood ratio test identified that the model log-likelihood was improved. We stopped this procedure when additional parameters failed to result in a significant likelihood ratio test [49]. This procedure gave us our final model for evaluation.

We evaluated the predictive potential of this final model through receiver operating curves (ROC) and k-fold cross validation test [49,50]. From the ROC we calculated the area under the curve (AUC). The AUC value ranges from 0.5–1 where 0.5 is random and 1 indicates perfect discrimination. Models with AUC values greater than 0.7 are considered fair and those with AUC values exceeding 0.9 are considered highly accurate [49]. For the k-fold cross validation test we generated 5 random subsets of the database each maintaining an 80:20 ratio of training to testing data. In this fashion we fit models using 80% of the dataset and validated the model using the remaining 20%. For each subset we developed a prediction, categorized it into 32 classes, and then simplified them into 10 bins [50]. We compared frequencies for each RSF bin between the training and the testing data for each subset and calculated Spearman’s rank correlation.

## Results

### Toad detection

Between 2012 and 2013, we detected 91 toads in the terrestrial landscape during the breeding season (Fig 1). Of these, one toad was recaptured in 2012 and then again in 2013 and so we removed the second recapture from the analysis leaving 41 relocated in 2012 and 49 in 2013. Of the 90 individuals, 13 were females, 77 were males, which was consistent with the sex ratio at tagging in the breeding pond (approximately 6 males per female). Toads were recaptured having travelled between 15 and 360m from the breeding pond. Our documented common toad recapture rate approached 9%. The residual body condition index was calculated using residuals of the linear regression of weight versus SUL [51]. The residual index of recaptured males did not differ significantly between capture in the pond versus later in the terrestrial environment (paired t-test, t<0.001, p = 1, df = 67), implying no loss of condition since tagging (pairs with missing values were excluded, females were excluded due to fluctuating weight dependent on whether or not they were carrying eggs).

### Resource selection function modelling

**Fig 2:**
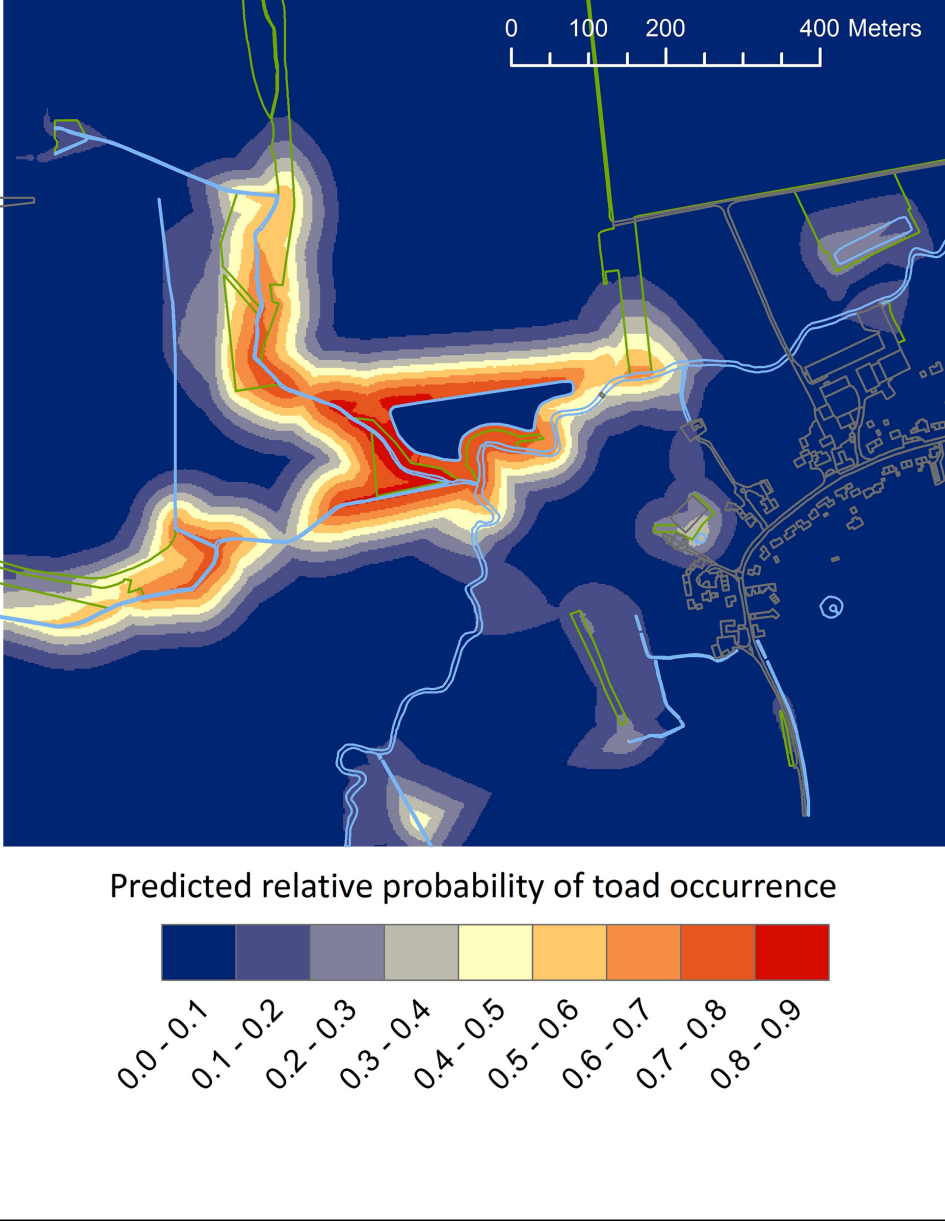
The predicted relative probability of common toad occurrence in Garford, Oxfordshire based on PIT tag data collected between 2012 and 2013. Habitat characteristics outlined in green = wooded habitat, grey = urban, blue = water bodies.

**Fig 3:**
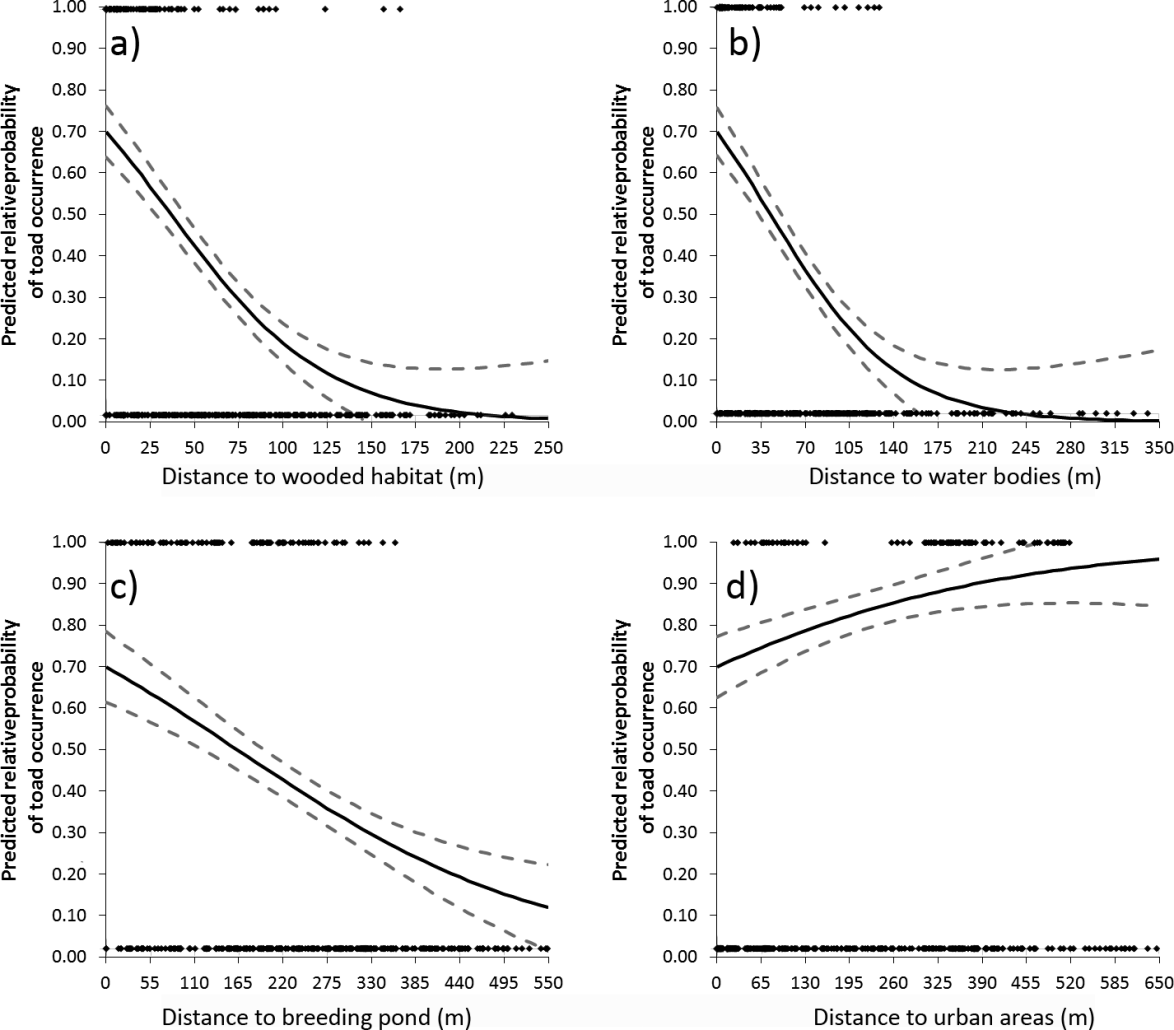
Mixed logistic regression functions for the environmental features in the final model predicting the relative probability of toad occurrence in Garford, Oxfordshire based on PIT tag data collected between 2012 and 2013. Dashed lines show the 95% confidence intervals of each estimate.

In relation to the 90 used locations that we identified via PIT tag relocation, we generated a total of 275 available locations (124 in 2012 and 152 in 2013). Our collinearity matrix revealed that distance to edge habitat was highly correlated with both distance to wood (|r| = 0.69, P < .001) and distance to water (|r| = 0.71, P < .001). We removed distance to edge habitat from consideration because it was the weakest predictor of the three. Distance to arable lands did not significantly improve model fit (*X*^2^ = 1.63, *P* = 0.20) and was not retained for analysis. The final model predicting the relative probability of common toad occurrence featured distance to wood, distance to water bodies, distance to pond, and distance to urban areas. This model yielded a prediction (Fig 2) that performed far better than average (area under the ROC curve = 0.891) with k-fold cross validation tests revealing that the training and testing data were well correlated (average r_s_ = 0.638). The predictive map developed in ArcMap from the final model revealed that the predicted relative probability of toad occurrence was greatest in habitat bordering the western portion of the breeding pond (Fig 2). The lowest predicted relative probability of toad occurrence was associated with habitat in the human-dominated landscapes surrounding the breeding pond (Fig 2). Specifically, the predicted relative probability of toad occurrence decreased with distance away from wooded areas (Fig 3A), water bodies (Fig 3B), and the breeding pond (Fig 3C). Relative probability of toad occurrence declines quickly with increasing distance to water bodies and wooded habitats (approximately zero probability at 245m and 200m respectively) and more steadily for distance to the breeding pond. Conversely, the predicted relative probability of toad occurrence increased in habitat at a greater distance from urban areas (Fig 3D).

## Discussion

Common toad probability of occurrence was positively associated with the presence of nearby wooded habitat, declining with increasing distance from these environmental features. This observation is consistent with previous research where toad population size was positively associated with presence of woodland, and hedgerows within the terrestrial habitat matrix [20], terrestrial distribution was positively associated with forest cover [17,20,52] and amphibian and reptile species richness positively correlated with proportion of forest cover [30]. Within wooded habitats and in the areas nearby, common toads would have easy access to dense vegetation which would be more stable in temperature and so offer protection from desiccation or freezing, and may offer reduced predation pressure with more opportunities to hide. Positive selection for woodland [18,20,52] (particularly broadleaved) and rough grassland in the terrestrial environment [53,54] is well documented in other studies. The western toad (*Anaxyrus boreas*) similarly used forest, wet scrubland and grasslands as foraging habitat in Canada [55].

Distance from water bodies had a marked effect on the relative probability of occurrence of common toads in this terrestrial environment. This result is likely due to the need for these amphibians to avoid desiccation. The results agree with Salazar [56] where colonization of field margins by toads in Oxfordshire was negatively associated with the distance from the nearest pond, with Atauri and de Lucio [57] who demonstrated importance of damp habitats in determining amphibian presence in the region of Madrid, Spain and Findlay and Houlahan [30] who showed a positive relationship between herpetofauna species richness and wetland area in Ontario, Canada. This finding highlights the potential effect of reduced availability of wet habitats, such as ponds [7], on the occurrence of water dependent species.

The population of toads in this study appear to be isolated as evidences by the probability of occurrence dropping steadily with increasing distance from the breeding pond, to approximately 10% at 550m (Fig 3C). This isolation may be due to intensive farmland causing habitat fragmentation to the North of the breeding pond, with urban habitat (buildings and roads) posing a barrier to the South East. As such, the results of this study should not be taken as representative for toads in all farmed landscapes. Though the habitats used in the analyses were chosen so as to be more widely comparable (i.e. woodland, water, urban) it is important to understand that barriers to migration (e.g. roads) may be limiting the choice of available habitat for the population studied. As in Van Buskirk [58], local habitat availability, as determined by the presence of barriers, is important in driving the landscape level effects of habitat on species distributions. Similarly Zanini et al. [33] demonstrated greater predictive power of models for habitat patch occupancy for toads when available habitat in a buffer zone took into account the presence of barriers. Furthermore, we would expect lower species richness of amphibians where road density is higher [30] and amphibians are also sensitive to habitat fragmentation, with reduced amphibian abundance recorded in riparian buffer strips when compared with intact forests [59].

Our results indicate that increased density of both wooded and wet habitats (e.g. through pond and ditch creation) in farmland should be expected to improve the utility of intensively farmed landscapes for the common toad during the terrestrial phase by reducing potential distance to these positively selected habitats. The resulting increased habitat heterogeneity is also likely to enhance species diversity in farmland habitats for a suite of other taxa [57,60,61]. Wooded habitat and water bodies important during the foraging period are also key in providing hibernacula for the winter, for example in western toads, hibernation sites were located in woodland stands and in vegetated buffers along watercourses [62]. Other temperate amphibian species have similar requirements for the habitat in which they move through and forage during the summer as they will also need to thermoregulate and avoid desiccation and predation. Increasing habitat heterogeneity would also increase landscape permeability by reducing the risk associated with larger movements through increasing the availability of favourable habitats. A review of the effect of habitat loss and fragmentation on amphibians by Cushman et al. [63] highlighted the crucial role of connectivity for persistence of populations.

We also found that common toads selected habitat in the vicinity of the breeding bond. This effect of distance from the breeding pond on toad probability of occurrence was expected as toads are only a moderately mobile species [40]. This does however, highlight the importance of increasing pond density to facilitate movement between breeding populations (of this and other water dependent species) at the landscape scale and to redress the dramatic loss of ponds witnessed in the last century [7].

The predicted relative probability of toad occurrence was negatively associated with urban areas suggesting higher resistance of these habitats and corroborating the findings of Hitchings and Beebee [64], Janin et al. [65] and Findlay and Houlahan [30]. The corresponding increased relative probability of occurrence on the western side of the breeding pond (Fig 1) highlights the importance of considering the configuration of the landscape [e.g. 65] rather than just the composition. Our results suggest that spatial variation in the urban footprint can affect the distribution of common toads in the terrestrial landscape. For instance, wooded habitat near to a water body was not widely selected by toads in our study when those habitats were located close to an urban area. Urban and agricultural adjacent land use has been shown to affect habitat quality (e.g. water quality and sediment load in water bodies) at large distances [34] and so may reasonably expected to alter suitability of otherwise favoured habitats and thus the probability of species occurrence. Though distance to arable farmland was not identified as an important predictor of toad occurrence, we did not recapture a single toad in cultivated fields, which accords with the negative association with arable farmland reported by Piha et al. [36] and avoidance of ploughed land described by Janin et al. [66] and intensive farmed habitats may have contributed to the isolation of this population.

This is the first instance in which a portable PIT antenna has been used to aid detection of a species in an open system over a large terrestrial area. The method was successful, facilitating collection of sufficient presence data points for detailed modelling without compromising the welfare of the study species and could be promising for the study of anurans in other systems. It would be interesting to determine whether individual toads exhibit the same level of fidelity to foraging and hibernation sites as they do to their breeding pond. This was not possible in the present study as all toads were recaptured during their resting periods and only one individual was recaptured in both years. Further work should look at small scale home ranges of uniquely identifiable toads over multiple years to determine the influence of different habitat types on common probability of occurrence and identify the spatial scale at which they are most important. However, insofar as the isolation of this population makes it difficult to infer the probability of toad occurrence in other farmed landscapes, we should also endeavour to study toads at a variety of breeding ponds in farmland, in order to make recommendations that are more widely applicable, as suggested by Cushman et al. [63] to create a conservation plan that can be implemented at the landscape scale.

We recommend that conservation action for the common toad be centred on the creation of suitable habitat to increase the density of wooded habitats and water bodies in farmed landscapes. In particular, it is important to provide suitable breeding ponds to reduce distance between breeding populations and so encourage a higher degree of mixing. Furthermore, design of habitat corridors to connect isolated populations should consider diverting them from urban areas where possible (it is possible to overcome pond fidelity with translocations to new suitable breeding ponds, e.g.[67]) and using woody vegetation and linear water bodies (such as ditches and streams), which are apt to be more attractive to moving common toads.

## Supporting Information

S1 Dataset Shapefile of all toad recapture locations. Excel file of all recaptured toads weight, length and calculated body condition index in the breeding pond and terrestrial environment. Raster files of distance to nearest edge, distance to the breeding pond, distance to the nearest water body and distance to the nearest woodland habitat. Shapefiles of surveyed 100m x100m squares in 2012 and 2013. STATA analysis and code for the final RSF model. Excel file of location, PIT code and habitat type for all recaptured toads. Shapefiles of the breeding pond, urban habitat, water bodies and woodland habitat within 500m of the breeding pond.
(ZIP)
