# Supplementary material for: Mapping the Relative Probability of Common Toad Occurrence in Terrestrial Lowland Farm Habitat in the United Kingdom
Source: PLoS One. 2016 Feb 3;11(2):e0148269. doi: 10.1371/journal.pone.0148269 (PMC4739741; doi:10.1371/journal.pone.0148269)
Supplement: S1 Dataset — Excel file of all recaptured toads weight, length and calculated body condition index in the breeding pond and terrestrial environment. Raster files of distance to nearest edge, distance to the breeding pond, distance to the nearest water body and distance to the nearest woodland habitat. Shapefiles of surveyed 100m x100m squares in 2012 and 2013. STATA analysis and code for the final RSF model. Excel file of location, PIT code and habitat type for all recaptured toads. Shapefiles of the breeding pond, urban habitat, water bodies and woodland habitat within 500m of the breeding pond. (ZIP) [file pone.0148269.s001.zip › SupportingInfo/STATA_Analysis_Toads.docx]

Final Model

. xtmelogit response dist_wood dist_water dist_lake dist_urban, || year:, covariance(unstructured) laplace

Note: single-variable random-effects specification; covariance structure set to identity

Refining starting values:

Iteration 0: log likelihood = -130.29252 (not concave)

Iteration 1: log likelihood = -128.42417

Iteration 2: log likelihood = -128.00078

Performing gradient-based optimization:

Iteration 0: log likelihood = -128.00078

Iteration 1: log likelihood = -127.68828

Iteration 2: log likelihood = -127.6875

Iteration 3: log likelihood = -127.6875

Mixed-effects logistic regression Number of obs = 366

Group variable: year Number of groups = 2

Obs per group: min = 165

avg = 183.0

max = 201

Integration points = 1 Wald chi2(4) = 69.34

Log likelihood = -127.6875 Prob > chi2 = 0.0000

------------------------------------------------------------------------------

response | Coef. Std. Err. z P>|z| [95% Conf. Interval]

-------------+----------------------------------------------------------------

dist_wood | -.0229473 .0050363 -4.56 0.000 -.0328182 -.0130764

dist_water | -.019867 .00557 -3.57 0.000 -.030784 -.00895

dist_lake | -.0051799 .0015117 -3.43 0.001 -.0081427 -.002217

dist_urban | .0035487 .0010933 3.25 0.001 .0014057 .0056916

_cons | .8432812 .3896757 2.16 0.030 .0795309 1.607031

------------------------------------------------------------------------------

------------------------------------------------------------------------------

Random-effects Parameters | Estimate Std. Err. [95% Conf. Interval]

-----------------------------+------------------------------------------------

year: Identity |

sd(_cons) | 1.82e-08 .218679 0 .

------------------------------------------------------------------------------

LR test vs. logistic regression: chibar2(01) = 0.00 Prob>=chibar2 = 1.0000

Note: log-likelihood calculations are based on the Laplacian approximation.

Final Predictive Equation

1/(1+Exp(-(0.8432812 + -0.0229473*("dist_wood ") + -0.019867*("dist_water ")+ -0.0051799*("dist_lake") + 0.0035487*("dist_urban"))))
